# Smoldering Waldenström macroglobulinemia coexisting with myelodysplastic syndrome: a rare case report and literature review

**DOI:** 10.3389/fonc.2026.1850617

**Published:** 2026-05-25

**Authors:** Huanyuan Wang, Shuai Tan, Yumeng Li, Wanling Sun

**Affiliations:** Department of Hematology, Xuanwu Hospital, Capital Medical University, Beijing, China

**Keywords:** acute myeloid leukemia, coexistence, monoclonal gammopathy, myelodysplastic syndrome, Waldenström macroglobulinemia

## Abstract

Waldenström macroglobulinemia (WM) is a low-grade non-Hodgkin lymphoplasmacytic lymphoma of the bone marrow, characterized by production of monoclonal immunoglobulin M (IgM) protein. Smoldering Waldenström macroglobulinemia (SWM) is asymptomatic and exhibits an increased risk of progression to WM. Myelodysplastic syndrome (MDS) arises from blood cancer in the hematopoietic stem cell compartment, featuring dysplastic changes in bone marrow and blood cells, along with cytopenias, including anemia, neutropenia, or thrombocytopenia. Their coexistence is extremely rare. We report a 74-year-old woman with IgM monoclonal gammopathy, anemia, and bone marrow dysplasia. Initially treated as WM, persistent cytopenias prompted reevaluation, leading to a revised diagnosis of SWM with MDS-IB-1 MDS with increased blasts-1. After six cycles of azacitidine, she achieved remission of MDS but rapidly progressed to AML and ultimately died. This case provides a key clinical lesson: persistent cytopenias during ibrutinib therapy were attributable to MDS progression rather than SWM, underscoring the importance of re-evaluation. Furthermore, it completely documents clonal evolution from 2.5% blasts (MDS with low blasts) to 6% blasts (MDS with increased blasts-1) and ultimately to AML (66% blasts), and it introduces the emergence of an FLT3-ITD mutation that rapidly drove the disease into AML even after the patient had achieved MDS remission. We also review the rare coexistence of WM and MDS/AML, and MGUS with MDS.

## Highlights

- Complete serial monitoring documenting clonal evolution from 2.5% blasts (MDS with low blasts) to 6% blasts (MDS with increased blasts-1) and ultimately to AML (66% blasts).
- Even after the patient achieved remission of MDS, the FLT3-ITD mutation emerged and rapidly drove the disease into AML.
- A clinical lesson that persistent cytopenias on ibrutinib were attributable to MDS progression rather than SWM, underscoring the importance of re-evaluation.

## Introduction

Smoldering Waldenström macroglobulinemia (SWM) is characterized by the presence of a serum monoclonal immunoglobulin M (IgM) value ≥3 g/dL and/or ≥10% bone marrow lymphoplasmacytic infiltration of the bone marrow but no evidence of end-organ damage such as anemia, bone lesions, hypercalcemia, renal insufficiency, or amyloidosis, with an increased risk of progressing to symptomatic WM (1). MDS represents a group of blood cancers that originates in the hematopoietic stem cell compartment, characterized by morphological dysplasia in bone marrow and peripheral blood cells, as well as cytopenias such as anemia, neutropenia, or thrombocytopenia. MDS is associated with an increased risk of developing acute myeloid leukemia (AML) (2). Patients typically present with symptoms of anemia, such as fatigue, and the condition should be suspected in older individuals with macrocytic anemia (3). Approximately 4 out of every 100,000 individuals are diagnosed with MDS each year (4). The co-occurrence of WM and MDS is extremely rare, with only a few cases reported in the literature. This case illustrates the rare co-occurrence of SWM and MDS in a 74-year-old female patient. The clinical presentation, diagnosis, differential diagnosis, molecular genetic characteristics, and treatment will be discussed. Additionally, the coexistence of WM with MDS/AML, as well as the coexistence of MGUS and MDS, has been reviewed.

## Case report

**Figure 1:**
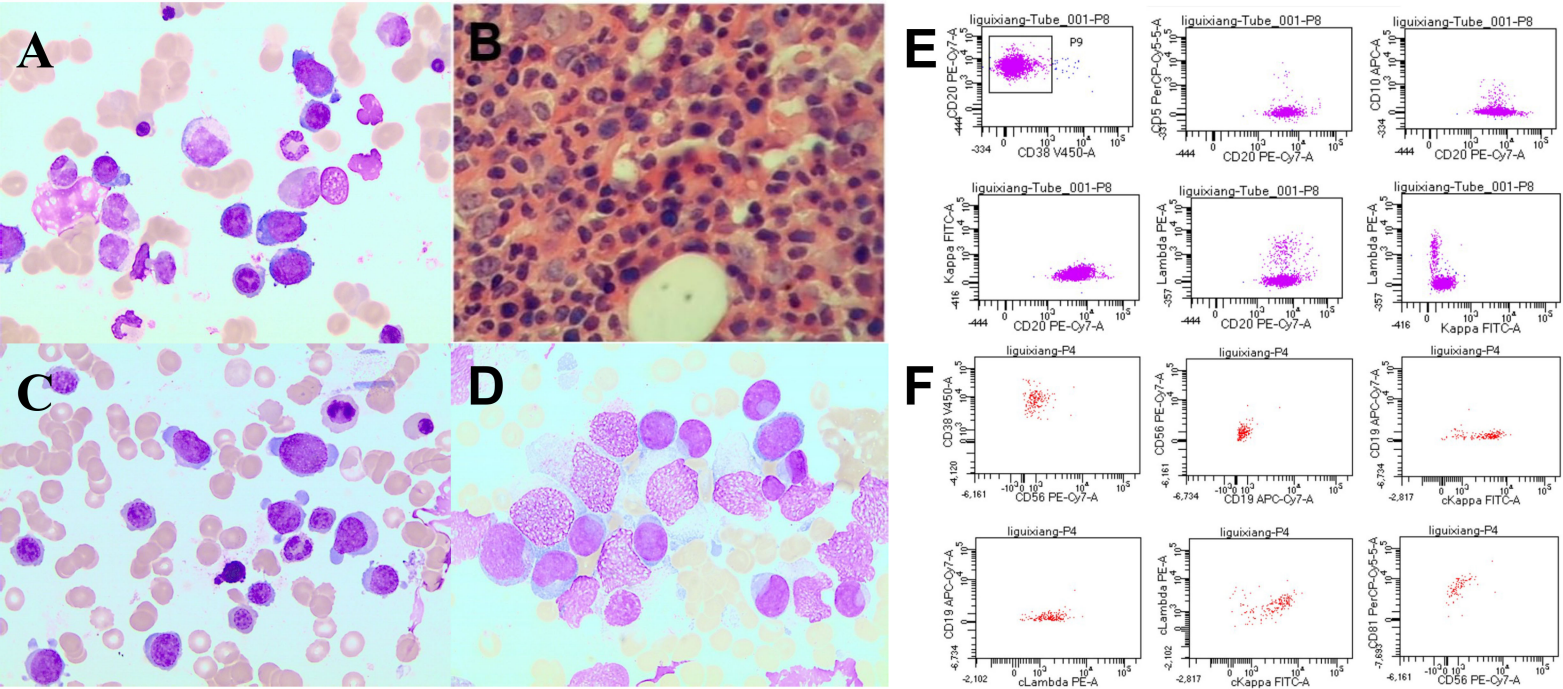
Bone marrow morphology smears at different stages and bone marrow flow cytometry indicating the coexistence of plasma cells and B cells. Phase 1: Diagnosed with WM. Phase 2: Diagnosed with concurrent SWM and MDS-IB-1. Phase 3: Transformation into AML. **(A)** Phase 1: The blast cells account for 2.5% of the total cell population. **(B)** Phase1: The bone marrow biopsy shows no presence of lymphoplasmacytic lymphoma cells. **(C)** Phase 2: The blast cells account for 6.0%, showing dysplastic hematopoiesis in the erythroid and megakaryocytic series. **(D)** Phase 3: The bone marrow smear exhibited significant proliferation activity, with primitive cells accounting for 66% of the total cell population. **(E)** CD19+ cells occupy approximately 3.1% of nuclear cells, expressing CD19, CD20, Kappa, and Lambda, suggesting aberrant monoclonal B lymphocytes. **(F)** CD45dim cells comprise about 0.1% of nuclear cells, expressing CD38, CD138, Kappa, and Lambda, indicating aberrant monoclonal plasma cells.

A 74-year-old female patient was admitted with a one-year history of fatigue and dizziness. Further workup detected macrocytic anemia (hemoglobin [Hgb] = 7.2 g/dL), a normal platelet count (platelets [Plt] = 150 × 10^9/L), proteinemia (IgM = 30.6 g/L), and monoclonal gammopathy (IgM Kappa = 3060 mg/dL). Serum protein electrophoresis revealed an M-spike, and immunoelectrophoresis indicated the presence of monoclonal IgM-kappa protein. Furthermore, bleeding, hemolytic, liver function, renal function, and autoimmune-related indicators were all normal (**Table 1**). Bone marrow examination revealed increased marrow blasts (2.5%), with dysplastic erythropoiesis and megakaryopoiesis. Howell-Jolly bodies, erythroblastic islands, and binucleate early erythroblasts are visible in the red cell system. Also, single round-nucleated micromegakaryocytes are visible (**Figure 1A**). Bone marrow biopsy revealed a small number of marrow cells, megakaryocytic dysplasia, single and double round-nucleated macrophages are visible, and scattered small lymphocytes and plasma cells (**Figure 1B**). The whole-body PET-CT revealed no hypermetabolic lesions, and no lymphadenopathy or splenic involvement was identified. Flow cytometry identified monoclonal B cells and plasma cells: monoclonal B lymphoid cells (CD19, CD20, CD79b) and monoclonal plasmocytes (CD38, CD138, CD45) (**Figures 1E, F**). Cytogenetic analysis revealed a normal 46XX karyotype. Myeloid genetic mutations include NRAS(40.40%) and NPM1(33.21%), while B-lymphoid genetic mutations include ARID1B(33.82%), NARS(41.13%), KMT2C(5.70%), MYD88(4.46%), CXCR4(1.24%).

**Table 1:** Laboratory data on admission.

Parameter	Value (reference range)	Parameter	Value (reference range)
Blood cell count		Biochemistry	
WBC, 10^9^/L	6.46 (4-10)	AST, IU/L	27 (8-40)
RBC, 10^12^/L	1.96 (3.5-5)	ALT, IU/L	10 (5-40)
Hb, g/L	72 (110-150)	LDH, IU/L	279 (109-245)
MCV, fL	101.5 (82-95)	ALB, g/L	29.26(35-55)
MCH, pg	36.7 (27-31)	BUN, mmol/L	3.81 (1.7-8.3)
Plt, 10^9^/L	150 (100-300)	Cre, μmol/L	60 (18-104)
		β2-MG, μg/mL Ca,mmol/L	2.60 (1.58-2.62) 2.12 (2.03-2.67)
Serological test		Urine test	
M protein, g/L	11.35 (0)	Urine protein, g/24h	0.11 (0-0.15)
IgM, g/L	30.60 (0.46-3.04)	Lambda light, mg/dL	<1.85 (0-1.85)
IgA, g/L	0.76 (0.82-4.53)	Kappa light, mg/dL	<5 (0-5)
IgG, g/L	8.53 (7.51-15.6)		
Complement C3, g/L	0.49 (0.79-1.52)		
Complement C4, g/L	0.02 (0.16-0.38)	Coagulation system	
Lambda light, mg/dL	336 (313-723)	APTT, s	38.7 (25-43.5)
Kappa light, mg/dL	3020 (629-1350)	PT, s	15.1 (11-15)
IEF	IgM (+)、kappa(+)	D-D, μg/mL	0.80 (0.01-0.5)
CRP, mg/L	11 (1-8)	INR	1.20 (0.8-1.2)
Autoimmune antibodies		Hematopoietic raw materials	
ANA	Negative	Vit B12, pg/mL	982 (180-914)
ACA	Negative	Folic acid, ng/mL	11.68 (3.1-19.9)
ANCA	Negative	Ferritin,ng/mL	492.7 (11-306.8)

WBC, white blood cell; RBC, red blood cell; Hb, haemoglobin; MCV, mean corpuscular volume; MCH, mean corpuscular haemoglobin; Plt, platelet; AST, aspartate transaminase; ALT, alanine aminotransferase; LDH, lactate dehydrogenase; LAB, Albumin; BUN, blood urea nitrogen; Cre, creatinine; β2-MG, β2-microglobulin; M protein, monoclonal protein; IgM, immunoglobulin M; IgA, immunoglobulin A; IgG, immunoglobulin G; complement C3, complement component 3; complement C4, complement component 4; Lambda light, lambda light chain; Kappa light, kappa light chain; IEF, Immunofixation electrophoresis; CRP, C-reactive protein; APTT, activated partial thromboplastin time; PT, prothrombin time; D-D, D-dimer; INR, international normalized ratio; ANA, Antinuclear antibody; ACA, Anticardiolipin antibody; ANCA, Anti-neutrophil cytoplasmic antibody; Vit B12, Vitamin B12.

Since MDS and MDS/MPN are diagnoses of exclusion, it was necessary to first evaluate this case for alternative causes of the observed bone marrow dysplasia and cytopenias, including bone marrow involvement by lymphoproliferative disorders (5). Despite no typical lymphoplasmacytic infiltration on initial bone marrow biopsy, SWM was diagnosed based on clonal B cells and clonal plasma cells by flow cytometry, elevated serum IgM (>3 g/L), and positive MYD88 and CXCR4 mutations. This morphologic and molecular discrepancy likely reflects focal or early disease, where a single biopsy misses heterogeneous neoplastic cell distribution. In light of symptom-related evidence such as anemia, she was treated with Bruton’s tyrosine kinase inhibitor (ibrutinib 420 mg daily). Concurrently, she received red blood cell transfusion, erythropoietin (EPO), and antibiotic treatments. However, continuous monitoring revealed progressive thrombocytopenia, with minimal change in hemoglobin levels. A repeat bone marrow examination showed an increase in marrow blasts (6.0%) and evidence of dyshematopoiesis (**Figure 1C**). The erythroid and megakaryocytic dysplasia are the same as in the previous bone marrow examination. A repeat cytogenetic analysis demonstrated an abnormal karyotype of 46,XX, dic(2;12)(q24;p12),+mar/46,XX. Flow cytometry analysis indicated except the coexistence of monoclonal plasma cells and monoclonal B cells, there was also a group of bone marrow primitive cells expressing CD34+, CD117+, and CD33 +. Acute leukemia-associated fusion genes were negative. Myeloid genetic mutations include FLT3-ITD(2.05%), NRAS(4.52%) and NPM1(3.97%). Because MDS is an exclusionary diagnosis, the presence of IgM, clonal B and plasma cells, with MYD88 and CXCR4 mutations, while without overt lymphoplasmacytic infiltration, which led to an initial diagnosis of WM. Ibrutinib lowered IgM but did not improve anemia, raising suspicion for MDS given baseline blasts of 2.5%. A repeat bone marrow examination showed 6% blasts, confirming smoldering WM and MDS with increased blasts-1 coexisting. For SWM, based on risk assessment, the patient was assessed as moderate risk and just required regular follow-up monitoring (6), so ibrutinib was discontinued. Intermediate risk by IPSS, very high risk by IPSS-R, and very high risk by WPSS, she began treatment with azacitidine (75 mg/m² subcutaneously on days 1–7 every 28 days).

**Figure 2:**
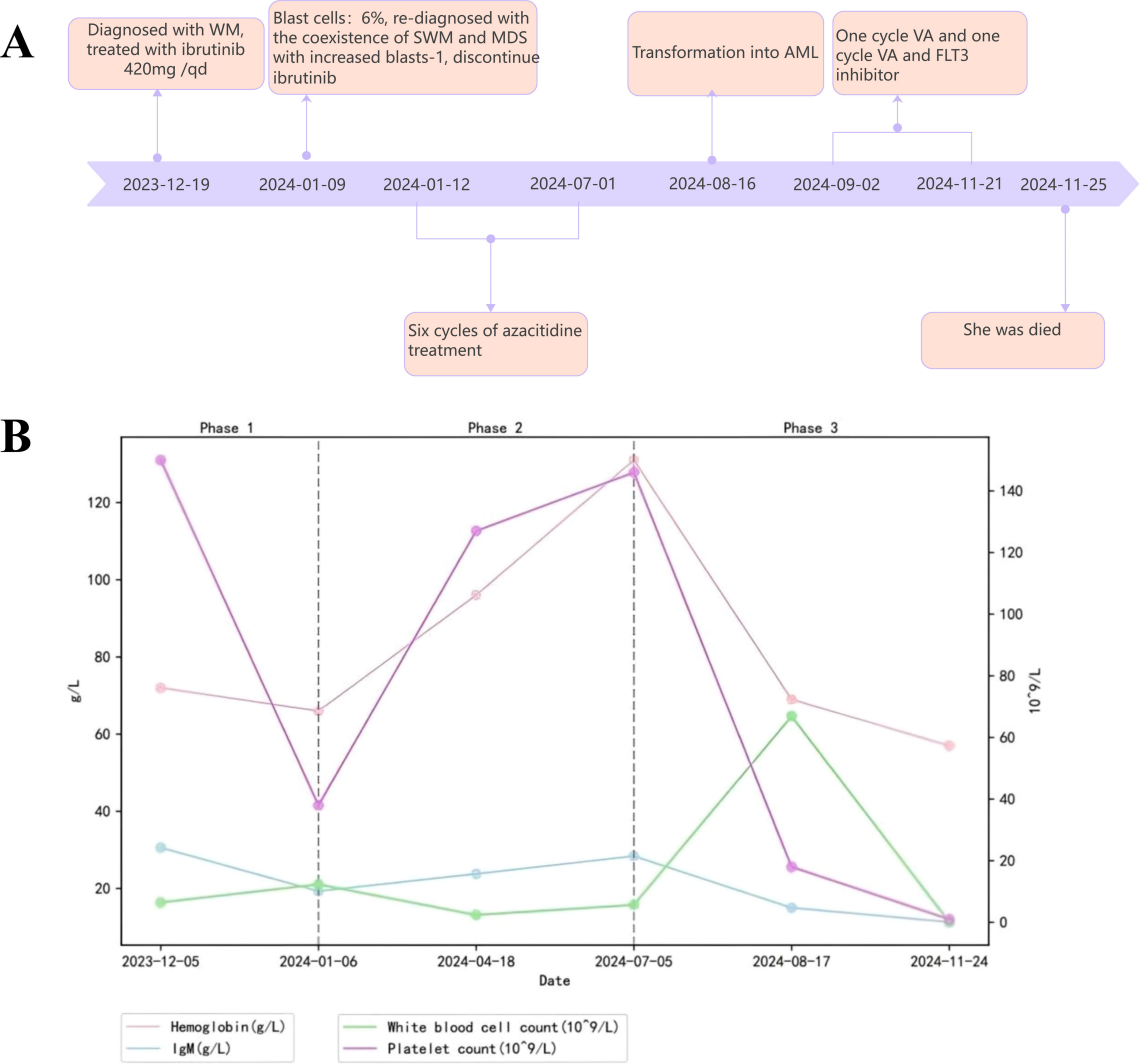
Treatment timeline illustrating the patient’s clinical course and treatment efficacy. **(A)** Treatment timeline during patient’s clinical course. **(B)** Phase 1: Diagnosed with WM. Phase 2: Diagnosed with concurrent SWM and MDS-IB-1. Phase 3: Transformation into AML. The changes throughout the entire disease process. Pink line: Hemoglobin, the changes in hemoglobin levels throughout the entire disease process Green line: White blood cell count, changes in white blood cell count throughout the entire course of the disease. Purple line: Platelet, changes in platelet count throughout the entire course of the disease. Blue line: IgM, Immunoglobulin M, changes in IgM levels throughout the entire course of the disease.

Six cycles of azacitidine were completed, with the last chemotherapy session occurring on July 7, 2024. The therapeutic response was assessed as complete remission (CR) of the MDS, as her hemoglobin and platelet counts returned to normal. But MDS with increased blasts-1 was associated with a high risk of progression to acute myeloid leukemia (AML) (7). One month later, on August 16, 2024, the repeated bone marrow aspiration showed 66% blast cells clustering (**Figure 1D**). While myeloperoxidase (MPO) expression was negative. Chromosomal examination was unsuccessful due to improper sampling. DNA sequencing of the bone marrow tissue confirmed mutations in the FLT3-ITD(91.02%), NRAS(44.53%), NPM1(43.50%). The diagnosis of transformation from MDS to AML was confirmed. The first monthly cycle of venetoclax in combination with azacitidine (VA) chemotherapy (VEN+AZA: Venetoclax 100 mg on day 1, 200 mg on day 2, 400 mg on day 3, azacitidine 75 mg/m² subcutaneously on days 1–7) began on September 2, 2024. Following one cycle of chemotherapy, a bone marrow examination revealed a decrease in marrow blasts (4.5%), however, there was no significant improvement in the complete blood count (Hgb = 6.0 g/dL, Plt = 27 × 10^9/L, WBC = 31.46 × 10^9/L), leading to an assessment of non-responsive. FLT3-ITD mutations indicated a poor prognosis (8). Subsequently, on September 29, 2024, she received a second course of VA and FLT3 inhibitor (Gilteritinib, 40mg/d1, 80mg/d2-d18, 40mg/d21-d40) chemotherapy treatment, but she had severe complications, including pneumonia, heart failure, and high propensity for bleeding, and ultimately died (**Figure 2**). Consistent with the NCCN Guidelines (Version 1.2024), the presence of an FLT3-ITD mutation categorizes AML into the adverse risk group. Gilteritinib is a category 1 NCCN recommendation for relapsed/refractory FLT3-mutated AML, but resistance, particularly secondary FLT3-ITD mutations, often limits its effectiveness leading to short survival as seen in this case.

## Patient perspective

Based on clinical observations, the patient experienced persistent fatigue during the disease course. Despite partial biochemical response to initial therapy, the lack of hematologic improvement prompted further evaluation. The patient tolerated treatment overall; however, disease progression was rapid, and complications occurred in the advanced stage.

## Discussion

### Literature review

The coexistence of WM and MDS/AML is extremely rare and has been limited only to case reports (**Table 2**). If we exclude cases of therapy-related MDS secondary to WM treatment, only three cases of coexistent WM and MDS at initial diagnosis have been reported to date. DeRosa et al. reported a 71-year-old male with MYD88-negative WM who developed a novel t(10;13) translocation and mutations including CXCR4 Q318 during the disease course (9). Motomura et al. reported a 55-year-old male initially diagnosed with MDS with IgM-MGUS who presented with unexplained fever and tested positive for the MYD88 mutation; after anti-LPL/WM therapy, his fever resolved, confirming that the fever originated from underlying LPL/WM rather than MDS (10). Schulze et al. reported a 60-year-old male with concurrent CMML (classified as MDS) and WM who received chlorambucil and cortisone but discontinued treatment due to poor compliance, leading to rapid deterioration and death three months later (11). Another case, published in 1995, reported a WM patient with a t(1;3)(p36;q21) translocation—an abnormality previously seen only in acute myeloid leukemia (AML) and myelodysplastic syndrome (MDS)—accompanied by bone destruction and hypercalcemia, suggesting transformation into IgM myeloma (12). Compared with the above cases, the present case describes a 74-year-old female who presented with IgM gammopathy, bone marrow dysplasia, and anemia at initial diagnosis. Because MDS is an exclusionary diagnosis, Smoldering Waldenström macroglobulinemia (SWM) was initially considered alone. However, after treatment with ibrutinib, the patient showed poor hematologic response. Repeat bone marrow examination revealed 6% blasts, confirming the coexistence of MDS and SWM. Through serial monitoring, we documented clonal evolution from 2.5% blasts to 6% blasts and ultimately to AML (66% blasts). Moreover, despite achieving MDS remission, the patient developed rapid AML transformation driven by FLT3-ITD, highlighting the aggressive potential of acquired FLT3-ITD in the context of synchronous MDS and SWM.

**Table 2:** Summary of WM coexistence with MDS/AML.

DOI	Author year	Age sex		Diagnosis	Mutation	Karyotype	Treatment	Outcome
10.3390/curroncol29070363.	PeterA DeRosa 2022	71y male		WM+MDS (concurrent)	Initial: PCR negative FIsh negative Later: CXCR4.et.al positive	Initial: 46,XY Later:del(20)(q11.2q13.1) Last:t(10;13)(p13;q22)	Lenalidomide, Azacitidine, Rituximab, Ibrutinib +Obinutuzumab, Reduced-dose Bendamustine + Rituximab	Died
10.1002/ccr3.5372.	Yotaro Motomura 2022	55y male		Initial: MGUS+MDS (concurrent) Later: WM+MDS (concurrent)	MYD88 Positive	none	Bortezomib+dexamethasone,+rituximab,	PR temperature normal
Waldenström’s macroglobulinemia with the AML/MDS-associated t(1;3)(p36;q21)	B Johansson 1995	56y female		WM	none	t(1;3)(p36;q21)	Chlorambucil, Cyclophosphamide + Vincristine + Prednisone, Cyclophosphamide + Vincristine + Mitoxantrone + Prednisone, Interferon-alpha	Progression to IgM myeloma
10.1007/BF00184549.	R Schulze 1992		60y male	Initial: WM+MDS (concurrent) Later: WM+AML	none	none	chlorambucil + cortisone	Died Progression to AML
10.1159/000530328	Shreyas Kalantri 2023		74y female	WM+t-MDS	none	7p-,17p-	Bortezomib + doxorubicin + bendamustine, R maintaine 15y, Bortezomib + Rituximab	VGPR, Dynamic monitoring
10.1093/jjco/hyi153.	Sang Joon Shin 2005		65y male	WM+t-MDS	none	5q-,12q-,-8	Chlorambucil Fludarabine	CR, Dynamic monitoring

The pathogenetic link between Waldenström macroglobulinemia (WM) and myelodysplastic syndrome (MDS) remains debated. It has been speculated that the cooccurrence of lymphoid and myeloid neoplasms may arise from a common clonal progenitor (13, 14). Conversely, the presence of double clonality is a possibility, wherein the lymphoid and myeloid clones facilitate each other’s selection and expansion in a vicious cycle (15). In our case, VAF analysis revealed nearly identical values for ARID1B (33.82%) and NPM1 (33.21%), suggesting a common progenitor origin. High VAFs of NPM1, NRAS, and ARID1B (33-41%) represent the dominant clone(s), whereas low VAFs of MYD88 (4.46%) and CXCR4 (1.24%) indicate that the WM clone comprised only a small fraction of bone marrow cells at diagnosis, explaining the absence of typical lymphoplasmacytic infiltration on initial biopsy. These findings favor to the likelihood of a model in which an ancestral hematopoietic stem cell acquired an ARID1B mutation, followed by lineage-specific branching into myeloid (NPM1/NRAS) and lymphoid (MYD88/CXCR4/KMT2C) subclones. Leleu et al. concluded that nucleoside analog therapy for WM, while effective, significantly increases the risk of transformation (4.7%) and t-MDS/AML (1.6%), with a very poor prognosis once t-MDS/AML occurs, with median survival 5 months (16). Kalantri et al. reported a 74-year-old female with relapsed Waldenström macroglobulinemia who developed therapy-related myelodysplastic syndrome (t-MDS) with complex karyotype abnormalities (del7q and del 17p) 15 years after receiving cladribine, bendamustine, and doxorubicin, highlighting the long-term risk of t-MDS following multi-agent DNA-damaging chemotherapy for WM (17). Our patient had not received any prior treatment at the time of initial diagnosis, thereby ruling out an iatrogenic cause. Unlike nucleoside analogs (Leleu et al.), BTK inhibitors such as ibrutinib carry a low risk of t-MDS, and the patient’s short exposure duration makes this etiology unlikely. A summary of several reported cases suggests that the prognosis of treatment for the coexistence of WM and MDS/AML is poor.

Although in this case the SWM did not transform into lymphoma but rather progressed from MDS to AML, insights can be drawn from literature on WM transforming into DLBCL. A 2026 study in Leukemia further demonstrated that BTKi-based immunochemotherapy regimens (such as ZR-CHOP) can significantly improve survival in WM patients, with particularly pronounced benefits for those with non-GCB subtype, double-expression, or extranodal involvement (18). Moreover, transformation events can occur at any stage of the disease, even during BTKi treatment. From a molecular perspective, the MYD88^L265P^ mutation may serve as the molecular basis for WM transformation, while mutations in CXCR4, TP53, and others drive clonal evolution (19). In this case, the B-lymphoid lineage harbored MYD88 and CXCR4 mutations, suggesting that similar molecular mechanisms may contribute to clonal evolution. Even though the disease ultimately progressed to myeloid leukemia rather than lymphoma, this highlights the genetic instability of smoldering WM during the latent phase and the inherent risk of disease progression.

MGUS and MDS are totally different clinical and pathologic entities. The underlying pathogenesis for the coexistence of MGUS and MDS remains to be determined. They may be connected to clonal heterogeneity, the bone marrow microenvironment, and immunosuppression (20, 21). Compared to healthy individuals, patients with MGUS have a significantly higher risk of developing MDS (22). After the diagnosis of MGUS, treatment is generally not necessary. Regular monitoring is sufficient (23). Regarding MDS, agents such as lenalidomide, azacitidine, and decitabine are FDA-approved for its treatment (24). Yan W et al. concluded that patients with MGUS and MDS-EB have a very poor treatment response, with many patients showing no response or rapid relapse (25). We reviewed some case reports of the coexistence of MGUS and MDS (**Table 3**). As described by Yan W et al. and others, these cases involve monoclonal gammopathy of undetermined significance rather than overt WM. Our patient presented with smoldering WM with confirmed MYD88 and CXCR4 mutations, which is a distinct lymphoplasmacytic lymphoma rather than a premalignant state. In summary, this case offers a crucial lesson—persistent cytopenias during ibrutinib therapy signaled MDS progression rather than refractory SWM, highlighting the need for re-evaluation. It also provides a complete longitudinal record of clonal evolution from MDS with low blasts (2.5% blasts) to MDS with increased blasts-1 (6% blasts) and ultimately to AML (66% blasts), and demonstrates that an acquired FLT3-ITD mutation can rapidly drive leukemic transformation even after MDS remission has been achieved.

**Table 3:** Summary of MGUS/SMM/MM coexistence with MDS.

Article	Author	Year	Diagnosis	Treatment	Outcome
Co-occurrence of monoclonal gammopathy and myelodysplasia: a retrospective study of fourteen cases	Yoshida Y	2014	12MGUS+MDS, 2MM+MDS	_	_
The effect of azacitidine therapy on the M protein of MDS patients with concomitant MGUS	Oka S	2018	8 MGUS+MDS	Azacitidine 75 mg/m^2^/d1-d7	8 Partial Remission
Coexistence of plasma cell neoplasia and myelodysplastic syndrome with excess blasts: case reports and literature review	Yan W	2021	2MGUS+MDS-EB-1, 12MM+MDS-EB-1/-2	_	_
Co-occurrence of myeloid neoplasm and plasma cell neoplasm	Jum'ah H	2022	SMM + MDS-EB-2	_	_
Successful treatment with bortezomib for refractory fever associated with myelodysplastic syndrome with underlying lymphoplasmacytic lymphoma	Motomura Y	2022	IgM MGUS+MDS	Bortezomib(1.3 mg]/m2 on days 1, 4, 8, and11, Dexamethasone 40 mg /body on days 1, 4, 8, and 11, and Rituximab 375 mg/m2 on day 11	Partial Remission
Rare Pattern of Myelodysplastic Syndrome (MDS) with Serum Monoclonal Immunoglobulin: Case Report	Yating Lin	2023	MGUS+MDS	Thalidomide (75 mg daily), dexamethasone (3 mg daily), and danazol (200 mg twice daily)	Stable
Co-occurrence of High-Risk Myelodysplastic Syndrome With a Complex Karyotype/TP53 Mutation and IgG Lambda Monoclonal Gammopathy of Undetermined Significance	Mir M	2024	MGUS+MDS 5q-	Decitabine, cedazuridine Bone marrow transplant	_

## Conclusion

This case presents a patient with IgM monoclonal gammopathy, anemia, and bone marrow dysplasia. Initially diagnosed with WM, she was treated with ibrutinib, however, her anemia persisted, and the number of blasts increased. The diagnosis was revised to SWM and MDS with increased blasts-1. After six cycles of azacitidine, she achieved complete remission, but then rapidly progressed to AML within a month and ultimately died. In summary, this case highlights that in the presence of IgM monoclonal gammopathy accompanied by bone marrow dysplasia, the possibility of coexisting lymphoid and myeloid neoplasms should be carefully considered. Dynamic follow-up and repeated bone marrow evaluations are essential for establishing an accurate diagnosis. In addition, patients with coexisting SWM and MDS generally have a poor prognosis and demonstrate that an acquired FLT3-ITD mutation can rapidly drive leukemic transformation even after MDS remission has been achieved, underscoring the need for close monitoring and individualized treatment strategies. Besides, WM/MDS coexistence is rare, reported either as synchronous at diagnosis or therapy-related. No standard treatment exists, and prognosis is poor. Whether WM affects MDS survival remains unclear. In our study, based on the VAF values of the gene mutations, a common progenitor origin is strongly favored. Similar uncertainty exists for MGUS/MDS coexistence.

## Data Availability

The original contributions presented in the study are included in the article/supplementary material. Further inquiries can be directed to the corresponding author.
